# Editorial: Fighting fire with fire: Using non-pathogenic viruses to control unrelated infections

**DOI:** 10.3389/fimmu.2022.1046851

**Published:** 2022-10-06

**Authors:** Tibor Bakacs, Konstantin Chumakov, Rifaat Safadi, Imre Kovesdi

**Affiliations:** ^1^ Department of Probability, Alfred Renyi Institute of Mathematics; The Eötvös Loránd Research Network (ELKH), Budapest, Hungary; ^2^ Office of Vaccines Research and Review, Food and Drug Administration, Silver Spring, MD, United States; ^3^ Hadassah Medical School, Faculty of Medicine, Hebrew University of Jerusalem, Jerusalem, Israel; ^4^ Unleash Immuno Oncolytics, St. Louis, MO, United States

**Keywords:** COVID-19 vaccine, molnupiravir and paxlovid, interferon (IFN) system, double-stranded RNA (dsRNA), attenuated viruses, superinfection therapy (SIT), intentional viral coinfection, controlled field trials

During the first year of the largest vaccination program in history 55.9% of the global population received at least one dose, 45.5% two doses, and 4.3% have received a booster dose of a COVID-19 vaccine reducing total deaths by 63% (1). The problem is, however, that despite such huge efforts and spectacular results, vaccination alone will not be able to control the COVID-19 pandemic because of the rapid evolution of the severe acute respiratory syndrome coronavirus 2 (SARS-CoV-2) even in vaccinated human populations. Genome sequencing showed that SARS-CoV-2 is picking up two mutations per month. The high circulation of the Delta and Omicron variants aided by inequitable vaccine distribution and insufficient control measures in some wealthy countries provide ground for further evolution of SARS-CoV-2, which could thus become more severe or evade current vaccines by recombining with other coronaviruses. Circulation in animal reservoirs could also bring unexpected changes (2). Adding insult to injury, according to WHO, COVID-19 vaccine hesitancy is one of the top threats that inhibits global control of SARS-CoV-2 infections (3).

The approval of oral antivirals (molnupiravir and Paxlovid) was a new milestone in the fight against the pandemic. To counter the threat of resistant variants, however, new drugs are needed requiring significant public and private investment (4). The United States, for example, provided USD 1.2 billion for basic research on developing antivirals for seven virus families. An important reason for such high costs is that antivirals, in contrast to antibiotics, are “one drug for one bug” therapies. Since more than two-hundred viruses are known to infect humans, while treatments are available only against a few of them, in order to close the gap between drugs and bugs a paradigm shift is required. The host rather than the virus should be targeted. A tempting target is, for example, the interferon (IFN) system, which is the antiviral agent of the host. During viral infections expression of type I IFN genes are many times upregulated in infected cells. Since the IFN system can control most, if not all, virus infections in the absence of adaptive immunity, it was proposed that viral induction of a nonspecific temporary state of immunity could be therapeutically exploited to provide a strategy to control viral infections (5).

This *Research Topic* gives a comprehensive overview about the impact of using naturally or intentionally attenuated viruses, which are non-pathogenic to people, to control viral diseases, with particular emphasis on the molecular mechanisms driving IFN induction and/or any other processes associated with response to therapy. Kasman proposed here the therapeutic exploitation of rhinoviruses (RVs), which are not associated with serious illness, hospitalization or mortality. RVs could be engineered for the benefit of their human hosts to induce immunity not just to a specific rhinovirus serotype but to other more pathogenic respiratory viruses as well. Seo and Seong discussed that based on their safety and potential for interference, live attenuated vaccines (LAVs) could be used as an alternative for immediate mitigation and control of unexpected pandemic outbreaks before pathogen-specific therapeutic and prophylactic measures are deployed. Cox et al. demonstrated that signaling through the type I IFN receptor, RV-mediated priming in the respiratory tract reduced viral replication, inflammation, and tissue damage of a pulmonary coronavirus infection preventing coronavirus mortality in mice. Stapleton reviewed “human pegivirus 1” (HPgV-1) infection (formerly known as HGV/GBV-C infection), which is common and may cause persistent infection in humans but the virus does not directly cause any known disease state. Importantly, several studies found that HPgV-1 infection was associated with prolonged survival in people living with HIV. The immunomodulatory effects of HPgV-1 are associated with beneficial outcomes in other virus infections including Ebola. Yagovkina et al. demonstrated in 1115 healthy volunteers (aged 18 to 65) that a single dose of bivalent Oral Poliovirus Vaccine (bOPV) significantly reduced the number of laboratory-confirmed cases of COVID-19 in the vaccinated group compared to placebo, consistent with the original hypothesis that LAVs induce non-specific protection against off-target infections. Therefore, bOPV could be used to complement specific coronavirus vaccines, especially in regions of the world where the vaccines are unavailable, and as a stopgap measure for urgent response to future emerging infections.

The translation of the ‘fighting fire with fire’ idea is perhaps closest to regulatory approval in the viral superinfection therapy (SIT) project, which is an *intentional* viral coinfection strategy (6). SIT administers a nonpathogenic attenuated double-stranded RNA (dsRNA) vaccine virus drug candidate, the infectious bursal disease virus (IBDV) serotype R903/78 that activates the IFN-genes from within cells. SIT has already been demonstrated to be safe and effective against five different families of viruses. SIT was effective against hepatitis A virus in marmoset monkeys, hepatitis B and C virus in 20/22 acute and 2/2 decompensated patients, SARS-CoV-2 virus in 5 acute patients, and herpes zoster virus in 1 patient. The therapeutic power of IBDV superinfection was demonstrated in a severe herpes zoster ophthalmicus infection, where disease duration was reduced from 3 – 5 weeks to a few days. The IBDV R903/78 drug candidate is simple to manufacture and easy to administer orally in an outpatient setting, its expected cost will be affordable even in resource-limited countries.

The feasibility of ‘fighting fire with fire’ strategy, using live attenuated viruses to control pathogen viruses, had already been validated by large controlled field trials involving more than 300 thousand people during three seasonal outbreaks of influenza and other associated acute respiratory infections with IFN-inducing live enteroviral vaccine (LEV) strains in the former Soviet Union (7). Administration of LEV provided protection and treatment against influenza and acute respiratory infections. Repurposing of existing infectious disease vaccines for the stimulation of anti-tumor immune responses was also proposed recently in this journal (8). Data supporting cancer therapeutic use was found for 16 infectious disease vaccines. In fact, focusing on the use of vaccines ‘as is’, may represent a cost-effective strategy to bring new therapeutic options to patients but requires special care in terms of clinical development and regulatory requirements.

## Author contributions

All authors listed have made a substantial, direct, and intellectual contribution to the work and approved it for publication.

## Conflict of interest

Author IK was employed by company Unleash Immuno Oncolytics. Authors TB and IK are shareholders of HepC, Inc.

The remaining authors declare that the research was conducted in the absence of any commercial or financial relationships that could be construed as a potential conflict of interest.

## Publisher’s note

All claims expressed in this article are solely those of the authors and do not necessarily represent those of their affiliated organizations, or those of the publisher, the editors and the reviewers. Any product that may be evaluated in this article, or claim that may be made by its manufacturer, is not guaranteed or endorsed by the publisher.
